# TO/TMMP-TMTGE Double-Healing Composite Containing a Transesterification Reversible Matrix and Tung Oil-Loaded Microcapsules for Active Self-Healing

**DOI:** 10.3390/polym11071127

**Published:** 2019-07-02

**Authors:** Nan Zheng, Jie Liu, Wenge Li

**Affiliations:** 1 Shaanxi Key Laboratory of Catalysis, School of Chemical and Environmental Science, Shaanxi University of Technology, Hanzhong, Shaanxi 723001, China; 2 School of Materials Science and Engineering, Shaanxi Key Laboratory of Catalysis, Shaanxi University of Technology, Hanzhong, Shaanxi 723001, China; 3 Merchant Marine College, Shanghai Maritime University, Shanghai 201306, China

**Keywords:** TO-loaded microcapsules, thiol-epoxy matrix, dynamic transesterification, intrinsic-extrinsic double self-healing

## Abstract

Thermoset epoxies are widely used due to their excellent properties, but conventional epoxies require a complicated and time-consuming curing process, and they cannot self-healed, which limits their applications in self-healing materials. Extrinsic and intrinsic self-healing materials are applied in various fields due to their respective characteristics, but there is a lack of comparison between the two types of healing systems. Based on this, a thiol-epoxide click reaction catalyzed by an organic base was introduced to achieve the efficient preparation of thiol-epoxy. Furthermore, tung oil (TO)-loaded microcapsules were introduced into the thiol-epoxy matrix of dynamic transesterification to obtain a TO/TMMP-TMTGE self-healing composite with an intrinsic–extrinsic double-healing system. For comparison, a TMMP-TMTGE self-healing material with an intrinsic healing system was also prepared, which contained only thiol and epoxy curing chemistries. The effect of the core/shell ratio on the morphology, average particle size, and core content of TO-loaded microcapsules was studied. It was found that when the core/shell ratio was 3:1, the average particle size of the microcapsules was about 99.8 μm, and the microcapsules showed good monodispersity, as well as a core content of about 58.91%. The differential scanning calorimetry (DSC) results showed that the TO core was successfully encapsulated and remained effective after encapsulation. Furthermore, scanning electron microscopy (SEM), atomic force microscopy (AFM), tensile tests, and electrochemical tests were carried out for the two types of self-healing materials. The results showed that the TO/TMMP-TMTGE composite and TMMP-TMTGE material both had self-healing properties. In addition, the TO/TMMP-TMTGE composite was superior to the TMMP-TMTGE material due to its better self-healing performance, mechanical strength, and corrosion protection performance.

## 1. Introduction

Polymers are widely used as protective materials, however, the structure and properties of polymers are easily changed upon application of an external stress, light, heat, and so on, which can cause local damage or micro-cracks that eventually lead to failure [1,2]. The emergence of self-healing materials effectively solves the above problems, so that local damage or micro-cracks are healed, thereby prolonging the service life and expanding the application range of polymers [3,4,5]. Currently, self-healing materials are mainly classified as either extrinsic or intrinsic self-healing materials. In extrinsic self-healing materials, the healing agent is usually stored in microcapsules, hollow fibers, or microvascular networks prior to embedding in the polymer matrix, and the healing agent releases and heals when cracks form [6]. Commonly used healing reactions are the ring-opening metathesis polymerization of dicyclopentadiene in the presence of Grubbs’ catalyst [7,8,9], siloxane polycondensation assisted by organotin catalyst [10,11], anionic ring-opening polymerization of epoxy catalyzed by imidazole [12,13], and cationic chain polymerization of epoxy assisted by boron trifluoride diethyl etherate [14,15]. Other examples include free radical polymerizations, such as benzoyl peroxide-initiated free radical polymerization of styrene [16], oxygen-assisted free radical polymerization of linseed oil and tung oil (TO) [17,18,19], together with thiol-ene photoinitiated free radical polymerization [20,21]. Extrinsic self-healing materials do not require manual intervention for crack sensing and repair activation and are very effective for repairing microcracks. However, it is difficult to achieve self-healing multiple times, and the compatibility between the added substance with the matrix should also be considered.

Intrinsic self-healing materials are healed by reversible covalent bonds or reversible non-covalent bonds in the inherent system [22]. Examples of reversible covalent bonds include Diels–Alder [23,24,25], transesterification reversible [26,27,28,29], disulfide [30,31,32], acylhydrazone [33], alkoxyamine moieties [34], trisulfide carbonate [35], trimethylene oxide [36], and borate [37]. Reversible non-covalent bonds are usually hydrogen bonds [38,39], metal coordination bonds [40,41], π-π stacking [42], ionic bonds [43], and so on. An intrinsic self-healing system does not need to consider the compatibility of added substances within the matrix and can theoretically achieve healing multiple times. However, there are still problems, such as relying on external stimuli (mechanical force, light, heat, pH, etc.) to complete self-healing [44]. Furthermore, the self-healing efficiency is unsatisfactory, the strength and Tg of the materials need to be improved, and commercial applications are lagging.

Epoxy resins as thermosetting polymers are widely used in many fields due to their good dielectric properties, acid and alkali corrosion resistance, heat resistance, mechanical properties, and low shrinkage during curing [45]. However, the curing of conventional epoxy resins was usually assisted by curing agents, and it is a time-consuming and complicated process. In addition, thermosetting polymers cannot be self-healed [46], which limits their applications. In the present work, a thiol-epoxy matrix was efficiently prepared by a thiol-epoxide click reaction catalyzed by an organic alkali [47]. At the same time, an extrinsic self-healing system was combined with an intrinsic self-healing system for the first time, so as to form an intrinsic–extrinsic double-healing system. In this system, TO-loaded microcapsules were embedded in a thiol-epoxy matrix of dynamic transesterification, thus forming the TO/TMMP-TMTGE self-healing composite. To compare the target TO/TMMP-TMTGE self-healing composite, a TMMP-TMTGE self-healing material containing only thiol and epoxy curing chemistries was prepared. In this study, the self-healing performance, together with the mechanical strength and corrosion protection performance of the two types of self-healing materials were investigated and compared by SEM, AFM, tensile tests, and electrochemical measurements.

## 2. Materials and Methods

### 2.1. Materials

Glycidyl methacrylate (GMA) was provided by J&K Scientifific Ltd., Beijing, China. Ammonium chloride (NH_4_Cl), potassium persulfate (KPS), sulfuric acid (H_2_SO_4_, 98%), hydrochloric acid (HCl, 37%), sodium hydroxide (NaOH), sodium chloride (NaCl), phenol, formaldehyde (37%), and resorcinol, were all provided by Tianjin Kemiou Chemical Reagent Co., Ltd., Tianjin, China. Tung oil (410 mPa s, 25 °C) was obtained from Guangzhou Hanhao Chemical Co. Ltd., Guangzhou, China. Trimethylolpropane tris (3-mercaptopropionate) (TMMP), trimethylolpropane triglycidyl ether (TMTGE), and 1,5,7-triazabicyclo[4.4.0]dec-5-ene (TBD, 98%) were all purchased from Sigma Aldrich Co. Ltd., Shanghai, China. All reagents were used without further purification.

### 2.2. Preparation of Hydrolyzed PGMA Particles

GMA (10.0 g) and KPS (0.2 g) were dissolved in water (200 g), then heated to 80 °C and allowed to react for 16 h, accompanied with a condenser and a mechanical stirrer. Then, H_2_SO_4_ (0.3 g) was added and reacted at 55 °C for 12 h. The products were washed with ethanol and distilled water, respectively, then centrifuged and dispersed in water, to obtain hydrolyzed PGMA particles.

### 2.3. Preparation of Pre-PF

Phenol (9.1 g), formaldehyde (12 g), sodium hydroxide (1 g), and water (11 g) were mixed and reacted at 70 °C for 6 h with magnetic stirring to form a prepolymer of phenol formaldehyde (pre-PF) solution. 

### 2.4. Synthesis of TO-Loaded Microcapsules and Preparation of TO/TMMP-TMTGE Self-Healing Composite

**Figure 1:**
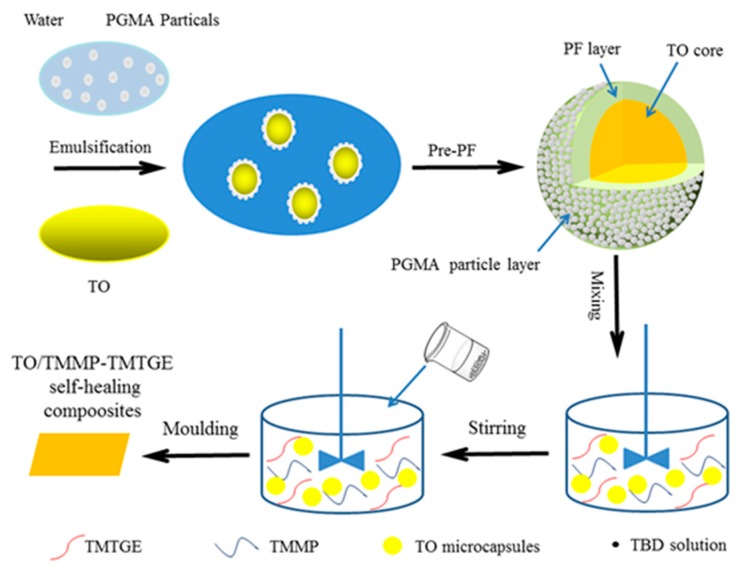
Preparation process of TO/TMMP-TMTGE self-healing composite.

Hydrolyzed PGMA stabilizers were dispersed in water, at a mass concentration of 2 wt% in the water phase. Subsequently, pre-PF (14 g) was added, and the pH of the system was adjusted to 5.0 using 3.7 wt% HCl solution. Afterward, resorcinol (1 g), ammonium chloride (1 g), and a given mass of TO were added, and the mixture was heated to 65 °C at a stirring speed of 600 rmp. After 10 min, the pH of the system was adjusted to around 0.5 using a 3.7 wt% HCl solution. The reaction was allowed to proceed for 1 h, then the microcapsule-encapsulated TO core was synthesized by the self-assembly of PGMA particles and in-situ polymerization of pre-PF. They were then washed three times with distilled water and air-dried at room temperature for 48 h. Detailed synthesis conditions for the TO-loaded microcapsules are listed in Table 1, and the synthetic procedure of TO-loaded microcapsules is displayed in Figure 1. 

**Scheme 1:**
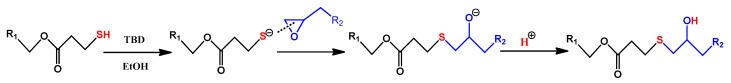
Schematic diagram of thiol-epoxide click reaction catalyzed by TBD.

TMTGE (2.0 g), TMMP (2.65 g), and TO-loaded microcapsules with a core/shell ratio of 3:1 (0.47 g) were mixed via magnetic stirring, and then TBD solution (0.046 g TBD, 0.7 mL ethanol) was added. After stirring for a short time, the mixture was poured into a mold and cured after a few minutes at room temperature, to eventually obtain the TO/TMMP-TMTGE self-healing composite. The preparation process mentioned above is also presented in Figure 1. The mechanism of the thiol-epoxide click reaction used to form the thiol-epoxy matrix is shown in Scheme 1. The TMMP-TMTGE self-healing materials containing only thiol and epoxy curing chemistries were both prepared according to the above process.

### 2.5. Characterization

Fourier-transform infrared spectra (FTIR) were recorded on an FTIR spectrometer (Bruker Tensor 27, Selb, Germany). Size distributions and average diameters of TO-loaded microcapsules were measured by a laser particle size analyzer (Beckman Coulter LS13320, Malvern, Chester, PA, USA). The morphology of microcapsules and self-healing materials were observed by an optical microscope (OM, 10XB-PC metallographic microscope, Shanghai, China), scanning electron microscope (SEM, FEI Verios G4, Hillsboro, OR, USA), and atomic force microscope (AFM, Bruker Dimension FastScan and Dimension Icon, Selb, Germany). Thermogravimetric analysis (TGA, TA Q50, Newcastle, DE, USA) was conducted from 35 to 800 °C at a heating rate of 10 °C min^−1^ under a nitrogen flow. The reactivity of the TO core was assessed by differential scanning calorimetry (DSC, Mettler Toledo DSC1, Zurich, Switzerland) at a heating rate of 10 °C min^−1^ from 0 to 225 °C under a nitrogen atmosphere. The shells of microcapsules were acquired by extracting the core materials.

An electrochemical workstation (Princeton ParStat4000, Knoxville, TN, USA) was used to obtain electrochemical impedance spectroscopy (EIS) spectra and Tafel plots. Using a conventional three-electrode cell, the prepared coatings were used as the working electrode with an area of 1 cm^2^ coated on a stainless steel surface. The reference electrode was a saturated calomel electrode, and the counter electrode was a platinum electrode [48]. The 3.5 wt% NaCl solution was used as the electrolyte, and the tests were carried out at room temperature. The frequency range was 10^−2^–10^5^ Hz for EIS tests, and the potential range was −0.5 to 1.5 V with a scan rate of 1 mV S^−1^ for the Tafel plots. The TO/TMMP-TMTGE composite and TMMP-TMTGE material were tested in their original states, after scratching and after healing (placed overnight at room temperature after scratching), respectively. 

The tensile strength of the dumbbell specimens of TO/TMMP-TMTGE self-healing composite and TMMP-TMTGE self-healing material were measured at 50 mm min^−1^ by a universal testing machine (Qingji Corporation QJ211S, Shanghai, China), according to standard ASTM D412.

## 3. Results and Discussion

### 3.1. Morphology, Size Distributions, and Average Diameters of TO-Loaded Microcapsules

**Figure 2:**
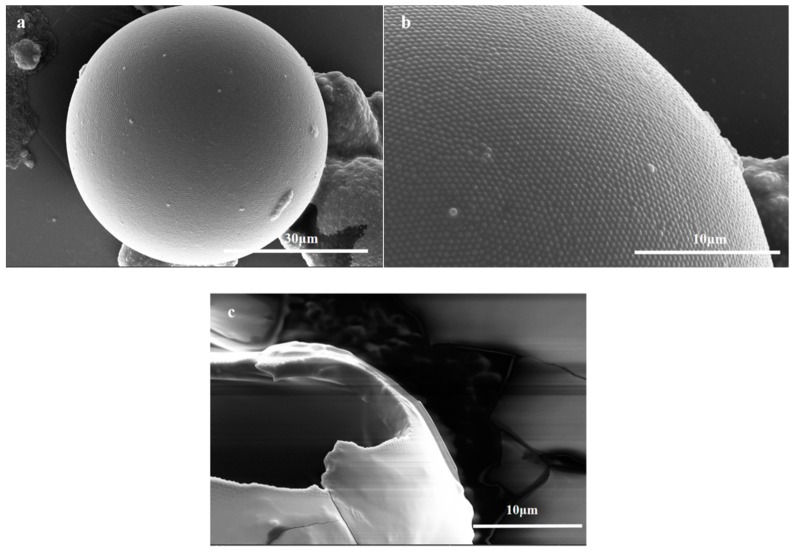
The SEM images of (**a**) TO-loaded microcapsules (core/shell ratio of 3:1), (**b**) the hydrolyzed PGMA particles coated on the microcapsules (core/shell ratio of 3:1), (**c**) a broken micocapsule (core/shell ratio of 3:1).

In this work, hydrolyzed PGMA particles were adsorbed at the oil-water interface of a Pickering emulsion to facilely and efficiently prepare microcapsules with excellent sphericity [49] (Figure 2a). The TO-loaded microcapsules exhibited spherical virus-like surface morphologies [50] via the compact self-assembly of PGMA particles on the surface of the microcapsule shells (Figure 2b), which can improve the anti-permeability and mechanical properties of the shells [6]. Furthermore, the hydroxyl groups of the outermost hydrolyzed PGMA particles layer promoted the dispersion and compatibility of TO-loaded microcapsules in the TMMP-TMTGE matrix. However, when core/shell ratios was 4:1, the excess TO core unencapsulated by the shell could deposit on the shell and form a cured film, which surrounded the capsule and covered the outermost PGMA particles (Figure S1). At the same time, a broken TO-loaded microcapsule revealed a smooth inner wall with a shell thickness of about 2.5 μm, as well as a smooth film covered by the PGMA particle layer (Figure 2c). It was indicated that the TO flowed out of the broken capsule and cured in air, showing that the TO core remained reactive and had a healing effect.

**Figure 3:**
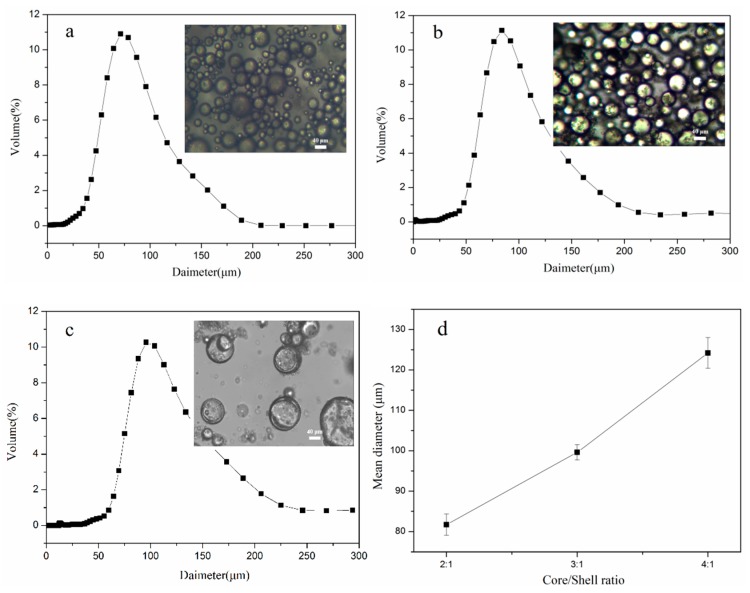
The size distributions and OM images of TO-loaded microcapsules with a core/shell ratio of (**a**) 2:1, (**b**) 3:1, and (**c**) 4:1, and (**d**) mean diameter of microcapsules with different core/shell mass ratios.

The size distribution and average diameters of microcapsules are shown in Figure 3. It can be seen that the particle sizes were normally distributed, with an average diameter of 99.8 µm and superior monodispersity for the microcapsules with core/shell ratio of 3:1 (Figure 3b). Moreover, the average diameter increased as the core/shell ratio increased (Figure 3d), and at a core/shell ratio of 4:1, the microcapsules adhered to each other (Figure 3c) due to the deposition of a large amount of unencapsulated TO core on the shells.

### 3.2. FTIR

**Figure 4:**
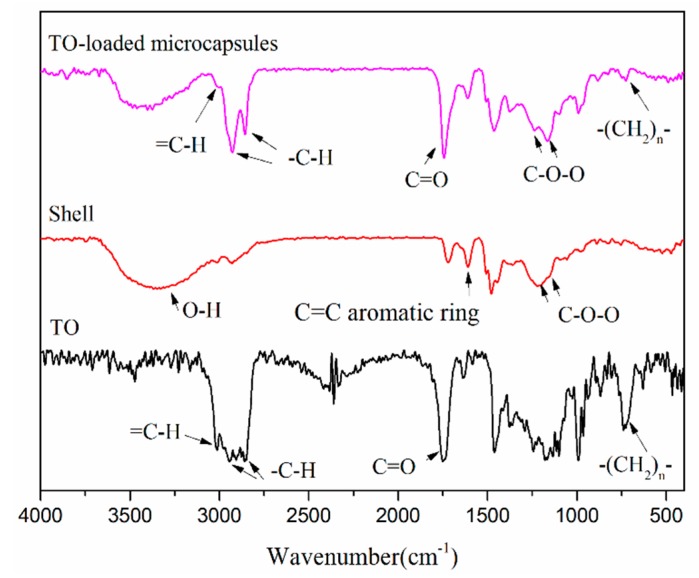
FTIR spectra of shell, TO-loaded microcapsules (core/shell ratio of 3:1) and TO core.

Figure 4 exhibits the infrared spectra of the TO core and TO-loaded microcapsules (core/shell ratio of 3:1), and the shells of the microcapsules. The spectra of the TO core and microcapsules shown in Figure 4 contained stretching vibration peaks of =CH at 3012 cm^−1^, stretching vibration peaks of C-H at 2927 cm^−1^ and 2855 cm^−1^, stretching vibration peaks of C=O at 1745 cm^−1^, and bending vibration peaks of -(CH_2_)_n_- (n ≥ 4) at 725 cm^−1^. These peaks indicated the TO core was successfully encapsulated and remained effective by retaining its original functional groups. Furthermore, the shell spectrum contained a wide stretching peak of O-H at 3368 cm^−1^, a stretching peak of C=C in aromatic ring at 1606 cm^−1^, a characteristic peak of phenolic hydroxyl group at 1385 cm^−1^, and stretching vibration peaks of ether bond C-O-C at 1259 cm^−1^ and 1163 cm^−1^. The presence of these peaks confirmed that the shell had both PF and PGMA components.

### 3.3. TGA

**Figure 5:**
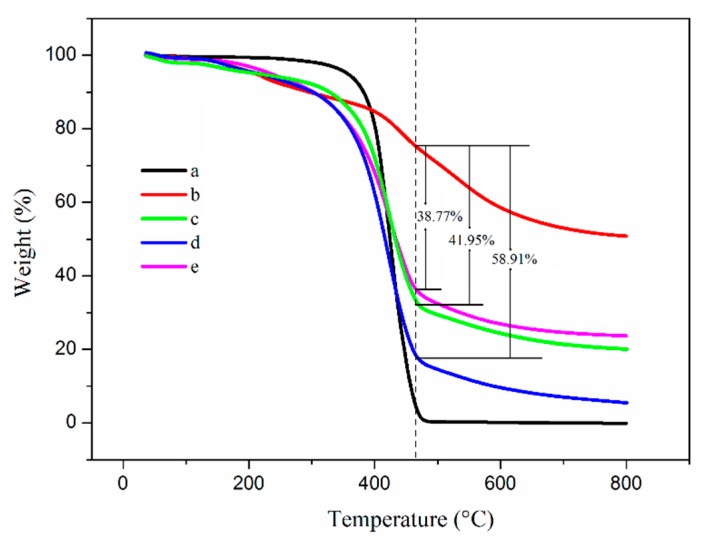
TGA curves of (**a**) TO, (**b**) shell, and TO-loaded microcapsules with core/shell ratios of (**c**) 2:1, (**d**) 3:1, and (**e**) 4:1.

The thermal performance and core content of TO-loaded microcapsules was evaluated by TGA measurements [51]. As shown in Figure 5, the initial decomposition temperature (defined as a weight loss of 5 wt%) of the TO core and shell material was about 357.1 and 210.5 °C, respectively. Furthermore, the initial decomposition temperature of the microcapsules increased as the amount of TO core increased. At a core/shell ratio of 4:1, the initial decomposition temperature reached a maximum of 235.2 °C. At the same time, when the core/shell ratio was 2:1 and 3:1, the temperature was 213.4 and 218.8 °C, respectively. The results indicated that the loading of TO increased the initial decomposition temperature of microcapsules, which was beneficial to the improvement of thermal stability of the microcapsules.

In addition, TO completely decomposed at 464.5 °C (defined as a residual 5 wt%). At this temperature, the weight of the shell was 75.38%, and the weight of the microcapsules with core/shell ratios of 2:1, 3:1, and 4:1, was 33.43%, 16.47%, and 36.61%, respectively. Therefore, the core content of the microcapsules was approximately 41.95%, 58.91%, and 38.77%, respectively (calculated in SI). It can be deduced that the microcapsules with a core/shell ratio of 3:1 had the highest core content, and at a core/shell ratio higher than 3:1, the excess TO core unencapsulated by the shell could deposit on the shell and form a cured film, thereby reaching a lower core content compared with the capsules with a core/shell ratio of 3:1.

### 3.4. DSC

**Figure 6:**
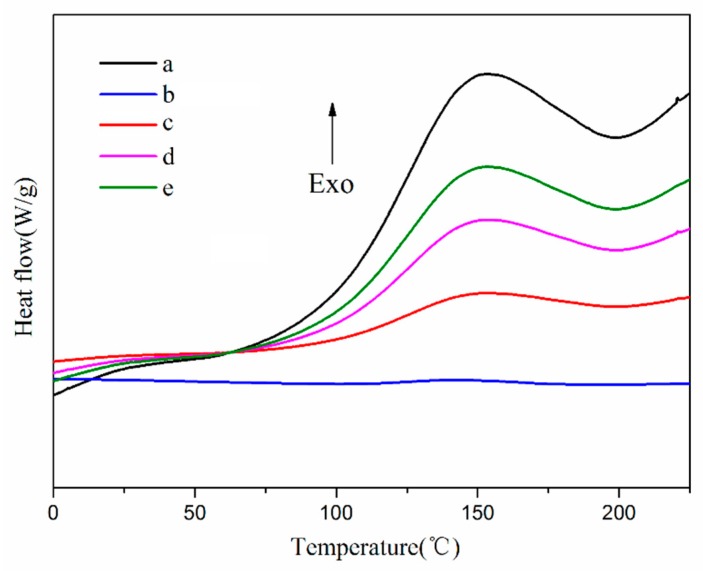
DSC heat traces of (**a**) TO, (**b**) shell, and TO-loaded microcapsules with core/shell ratios of (**c**) 2:1, (**d**) 3:1, and (**e**) 4:1.

The chemical reactivity of the encapsulated core material was a key factor in determining whether the microencapsulated core materials could self-heal, so the chemical reactivity of the core materials was evaluated by DSC [52,53]. As shown in Figure 6, the TO core showed a distinct exothermic peak at around 150 °C, which indicates that it reacted. In the DSC curves of the TO-loaded microcapsules with core/shell ratios of 2:1, 3:1, and 4:1, exothermic peaks also appeared near 150 °C. In contrast, no obvious exothermic peak was observed in the DSC curve of the shell, suggesting that TO was absent in the shell materials. Thus, TO was successfully encapsulated, and also remained as reactive as in its raw state.

### 3.5. Morphology of the Two Self-Healing Materials

**Figure 7:**
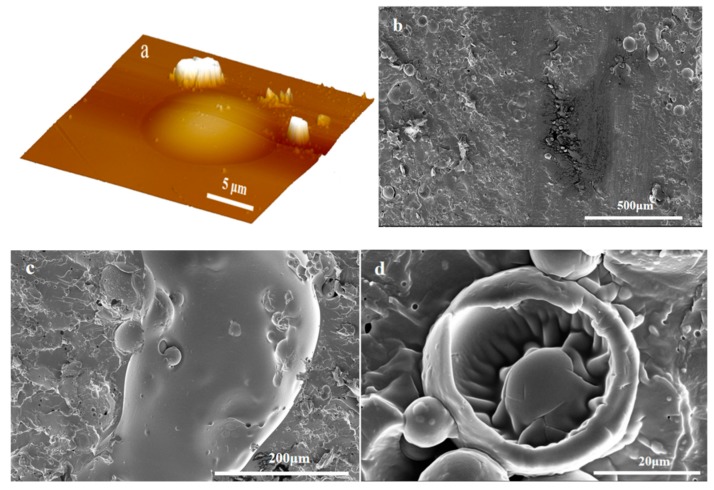
(**a**) AFM images of the surface, and SEM images of the fracture surface, (**b**) before and (**c**,**d**) after healing for the TO/TMMP-TMTGE self-healing composite.

**Figure 8:**
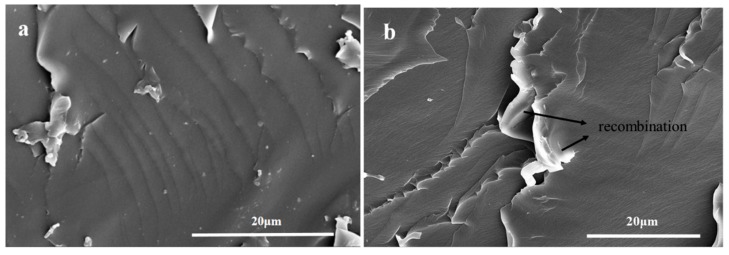
SEM images of the fracture surface (**a**) before and (**b**) after healing for the TMMP-TMTGE self-healing material.

**Figure 9:**
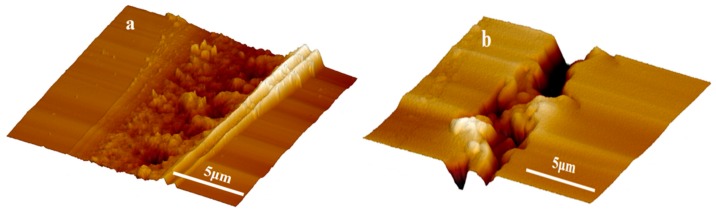
AFM images of scratch after healing for the (**a**) TMMP-TMTGE self-healing material and (**b**) TO/TMMP-TMTGE self-healing composite.

**Scheme 2:**
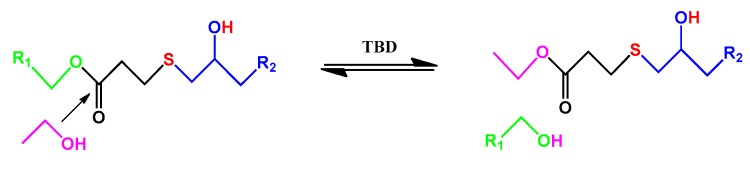
Schematic diagram of dynamic transesterification for the thiol-epoxy matrix.

It can be seen from Figure 7a that TO-loaded microcapsules with a core/shell ratio of 3:1 were well embedded within the TMMP-TMTGE matrix, and the fracture surface morphology of the TO/TMMP-TMTGE self-healing composite is shown in Figure 7b–d. In Figure 7b, a lubricated area is visible in the fracture surface after cutting the TO/TMMP-TMTGE composite, which was caused by the outflow of TO from the broken capsules. After being placed overnight at room temperature, the cured products distinctly appeared outside the ruptured TO-loaded capsules (Figure 7c,d), indicating that TO flowed out of the capsules and subsequently polymerized and solidified. Moreover, the fracture surface morphology of the TMMP-TMTGE self-healing material before healing and after healing are shown in Figure 8. After the fracture surface of the TMMP-TMTGE was healed (Figure 8b), the roughness increased noticeably compared with prior to healing (Figure 8a), which could be attributed to the recombination of chemical bonds in the transesterification reaction shown in Scheme 2. Finally, the TMMP-TMTGE material and TO/TMMP-TMTGE composite were scratched and then repaired, as shown in Figure 9. Some damaged areas on the TMMP-TMTG material were still not completely healed after being placed overnight (Figure 9a). However, the scratches were nearly completely filled after healing in the TO/TMMP-TMTGE composite, indicating it had a better healing effect compared with the TMMP-TMTGE (Figure 9b).

### 3.6. Tensile Strengths of the Two Self-Healing Materials

**Figure 10:**
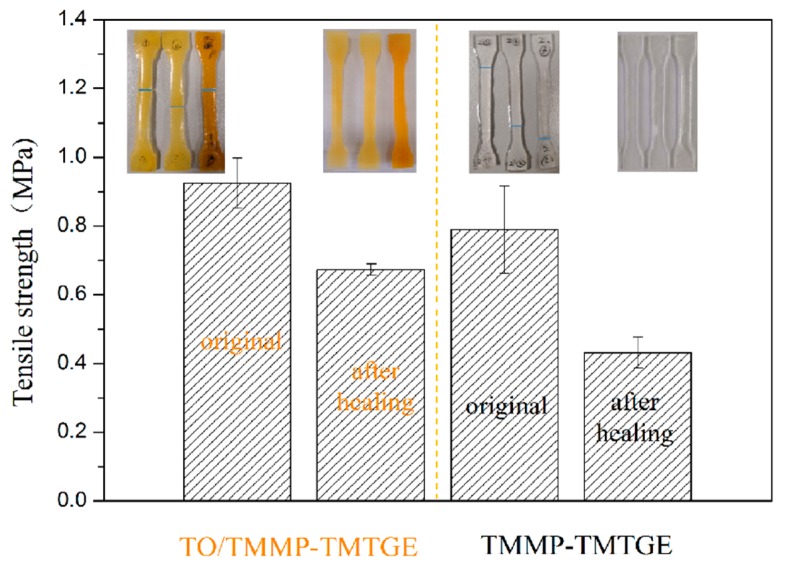
Tensile strengths of the TMMP-TMTGE self-healing material and TO/TMMP-TMTGE self-healing composite before and after healing.

As exhibited in Figure 10, the tensile strengths of the dumbbell specimens of the TO/TMMP-TMTGE self-healing composite and TMMP-TMTGE self-healing material were 0.92 and 0.79 MPa, respectively. After healing, the tensile strengths of the TO/TMMP-TMTGE and TMMP-TMTGE were 0.67 and 0.43 MPa, respectively. The photos in Figure 10 show that the pulled-apart portion of the dumbbell specimens in the two materials were reconnected after hot-pressing. After the TO/TMMP-TMTGE composite healed, its tensile strength reached 72.83% of its original state, and the tensile strength of the TMMP-TMTGE after healing reached 54.43% of its original state. This indicates that the filling of TO-loaded microcapsules in the thiol-epoxy matrix helped increase the strength of the matrix. Additionally, the TO/TMMP-TMTGE double-healing system demonstrated a better healing effect than the TMMP-TMTGE intrinsic healing system due to the double-healing mechanisms that included free radical polymerization of TO assisted by oxygen, together with the dynamic transesterification of the thiol-epoxy matrix. 

The TO/TMMP-TMTGE double-healing system demonstrated a better healing effect than the TMMP-TMTGE intrinsic healing system due to the double-healing mechanisms that included free radical polymerization of TO assisted by oxygen, together with the dynamic transesterification of the thiol-epoxy matrix. 

### 3.7. Electrochemical Properties of the Two Self-Healing Materials

**Figure 11:**
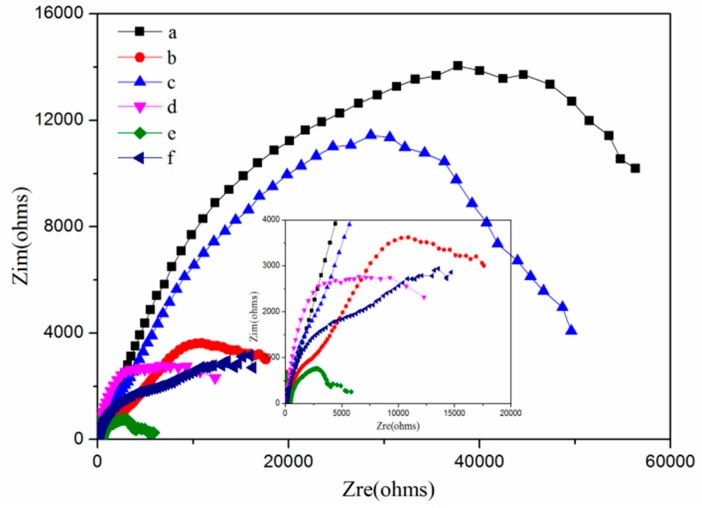
Nyquist plots of the TO/TMMP-TMTGE self-healing coating material (**a**) in the original state, and after (**b**) scratching and (**c**) healing, and the TMMP-TMTGE self-healing coating material (**d**) in original state and after (**e**) scratching and (**f**) healing.

**Figure 12:**
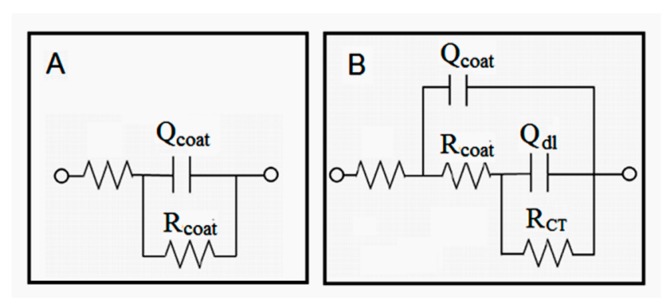
Equivalent circuit models for the (**A**) TO/TMMP-TMTGE and (**B**) TMMP-TMTGE self-healing materials.

EIS measurements were used to evaluate the self-healing performance of the TMMP-TMTGE self-healing material and TO/TMMP-TMTGE self-healing composite, as well as their corrosion protection performance. Nyquist plots of the two self-healing coating materials in their original states, after scratching and healing, are shown in Figure 11. The equivalent circuit diagrams shown in Figure 12 were used to simulate the electrochemical parameters. The equivalent circuit diagrams for the original state and after healing of the two coating materials were accorded with model A, and the diagrams of the two coating materials after scratching conformed to model B. In model A, the electrolytes did not reach the metal substrate, and the coating materials had better barrier properties. In model B, the two coating materials were scratched, the electrolytes reached the coating/metal interfaces, and the protective effect of the coatings was lost. 

In the models, *R*_coat_ is the coating resistance, *R*_CT_ is the charge transfer resistance, and *Q*_coat_ is the constant phase angle element related to the capacitance of the electrical double layer formed between the electrolyte solution and the surface of the coatings. *Q*_dl_ is the constant phase angle element related to the capacitance of the electrical double layer formed between the electrolyte solution and the surface of the base metal. These parameters correspond to the two models listed in Table S1.

As shown in Figure 11, the original state of the TO/TMMP-TMTGE had a higher arc radius than the original state of the TMMP-TMTGE, indicating that the filling of the TO-loaded microcapsules hindered penetration of the electrolyte. Moreover, the arc radius of the TO/TMMP-TMTGE after healing was three times the radius of the arc after scratching. However, after healing, the arc radius of the TMMP-TMTGE showed only a slight increase compared with after scratching. In addition, the arc radius of the TO/TMMP-TMTGE after healing was significantly larger than the radius of the TMMP-TMTGE after healing, which confirmed that the cracks were healed in both systems. Furthermore, the TO/TMMP-TMTGE double-healing system was superior to the TMMP-TMTGE intrinsic healing system, due to the combination of the double-healing and barrier effects. 

**Figure 13:**
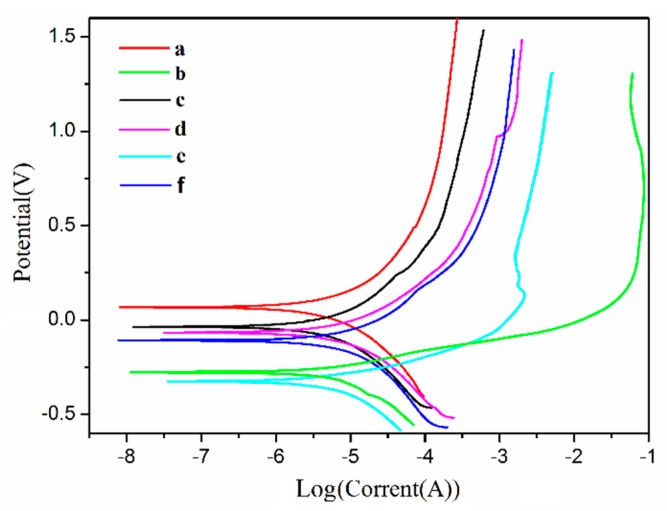
Tafel curves of the TO/TMMP-TMTGE self-healing coating material (**a**) in the original state, and after (**b**) scratching and (**c**) healing, and the TMMP-TMTGE self-healing coating material (**d**) in the original state, and after (**e**) scratching and (**f**) healing.

Potentiodynamic polarization curves were used to further characterize the self-healing performance as well as the corrosion protection performance of the two self-healing materials, as illustrated in Figure 13. The related parameters, corrosion voltage (*U*_corr_), and corrosion current density (*I*_corr_) were used to evaluate the corrosion resistance of the materials, as shown in Table S2. Figure 13 and Table S2 show that the original state of the TO/TMMP-TMTGE system had a lower *I*_corr_ value and a higher *U*_corr_ value than that of the TMMP-TMTGE system in its original state. This indicates that the filling of the TO-loaded microcapsules helped reduce the corrosion rate of the thiol-epoxy matrix to obtain a better anticorrosive effect [54]. In addition, after scratching the two self-healing materials, the *I*_corr_ values were both larger than their original states, and the *U*_corr_ values were both lower than their original states. This explains the increase in the corrosion rate of the two self-healing materials after damage. 

In the two systems, the *I*_corr_ values after healing were both smaller than the *I*_corr_ values after scratching, and the *U*_corr_ values after healing were both larger than the *U*_corr_ values after scratching. Furthermore, the TO/TMMP-TMTGE had a lower *I*_corr_ value and a higher *U*_corr_ value after healing compared with the TMMP-TMTGE after healing. This further certified that the two systems each had a self-healing effect on the cracks. In addition, the TO/TMMP-TMTGE double-healing system had a better self-healing ability and anticorrosion effect, which is consistent with the EIS results.

## 4. Conclusions

The microcapsules loaded the TO core with a PGMA particle layer and phenolic shell formed in one step in a Pickering emulsion, via the self-assembly of PGMA particles and the in-situ polymerization of pre-PF. The study of the core/shell mass ratios of the TO-loaded microcapsules showed that when the core/shell ratio was 3:1, the microcapsules had a mean particle size of 99.8 μm, showed good monodispersity, and the core content was 58.906%. The DSC results showed that the TO core material was successfully encapsulated and remained effective. At the same time, TO-loaded microcapsules were introduced into the thiol-epoxy matrix to obtain a TO/TMMP-TMTGE self-healing composite with a double-healing system. Moreover, a TMMP-TMTGE self-healing material with an intrinsic healing system was also prepared as a comparison. The SEM, AFM, tensile tests, and electrochemical tests were carried out on the two self-healing materials. The results showed that the TO/TMMP-TMTGE composite and TMMP-TMTGE material both had self-healing properties. In addition, the TO/TMMP-TMTGE composite had superior self-healing performance, better mechanical strength, and improved corrosion resistance compared with the TMMP-TMTGE material.

## Figures and Tables

**Table 1:** Recipes of TO-loaded microcapsules synthesized with different core/shell mass rations.

Sample	Core/Shell Mass Ration	PGMA (g)	Water (g)	Pre-PF (g)	TO (g)	Resorcinol (g)	NH_4_Cl (g)
1	2:1	0.5	25	14	13.45	1	1
2	3:1	0.5	25	14	20.18	1	1
3	4:1	0.5	25	14	26.91	1	1

## Supplementary Materials

The following are available online at https://www.mdpi.com/2073-4360/11/7/1127/s1, Figure S1: The SEM images of (a), (b) TO-loaded microcapsules (core/shell ratio of 4:1) at different magnifications, The core content of the TO-loaded microcapsules was calculated based on the TGA data shown in Figure 5, Table S1: Electrochemical parameters for TO/TMMP-TMTGE and TMMP-TMTGE self-healing coating materials obtained from EIS curves, Table S2: Electrochemical parameters for TO/TMMP-TMTGE and TMMP-TMTGE self-healing materials from Tafel curves.
