# Supplementary material for: TO/TMMP-TMTGE Double-Healing Composite Containing a Transesterification Reversible Matrix and Tung Oil-Loaded Microcapsules for Active Self-Healing
Source: Polymers (Basel). 2019 Jul 2;11(7):1127. doi: 10.3390/polym11071127 (PMC6680642; doi:10.3390/polym11071127)
Supplement: Supplementary file 1 [file polymers-11-01127-s001.pdf]

Supporting Information

TO/TMMP-TMTGE Double-Healing Composite Containing a Transesterification Reversible Matrix and Tung Oil-Loaded Microcapsules for Active Self-Healing

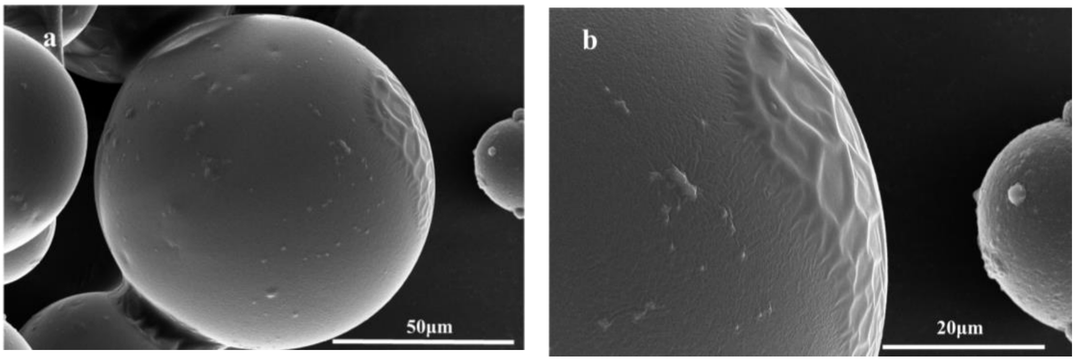

Figure 1. The SEM images of (**a**), (**b**) TO-loaded microcapsules (core/shell ratio of 4:1) at different magnifications.

The core content of the TO-loaded microcapsules was calculated based on the TGA data (Figure 5):

As for TO-loaded microcapsules, weight losses include whole core material and partial shell material. It can be seen from the TGA curve of TO core material, TO completely decomposed at 464.5 ℃ (defined as a residual 5 wt%). However, at this temperature, shells and microcapsules both have not finished decomposition. As a result, core mass fraction for each of microcapsules with different core/shell ratios can be calculated as weight fraction difference between shells and microcapsules at 464.5℃ approximately. By referring to Reviewer 52, 54, the core fraction of microcapsules of 2:1, 3:1, 4:1 was observed to be 41.95%, 58.91%, and 38.77%, respectively, which were marked in Figure 5.

The core content of TO-loaded microcapsules (W_c) was calculated by the following formula.

$$W_c = W_m - W_s$$

Where W_m, W_s is the weight fraction of microcapsules and shell when TO core is completely decomposed (464.5°C), respectively.

Table 1. Electrochemical parameters for TO/TMMP-TMTGE and TMMP-TMTGE self-healing coating materials obtained from EIS curves.

Sample	R_{coat} ($\Omega \cdot cm^2$)	Q_{coat} ($\Omega^{-1} \cdot cm^{-2} \cdot s^n$)	n_1	Q_{dl} ($\Omega^{-1} \cdot cm^{-2} \cdot s^n$)	n_2	R_{cT} ($\Omega \cdot cm^2$)
TO/TMMP-TMTGE (Original state)	6.43×10^4	1.115×10^{-5}	0.4868			
TO/TMMP-TMTGE (After scratching)	1.341×10^4	4.091×10^{-5}	0.277	1.697×10^{-5}	0.8145	1.448×10^4
TO/TMMP-TMTGE (After healing)	6.243×10^4	1.643×10^{-5}	0.4261			
TMMP-TMTGE (Original state)	2.059×10^4	1.556×10^{-5}	0.7126			
TMMP-TMTGE (After scratching)	6.440×10^3	5.936×10^{-4}	0.8341	9.661×10^{-5}	0.4299	4.891×10^3
TMMP-TMTGE (After healing)	2.012×10^4	3.39×10^{-5}	0.3205			

Table S2 Electrochemical parameters for TO/TMMP-TMTGE and TMMP-TMTGE self-healing materials from Tafel curves

Sample	U_{corr}（V）	I_{corr} (A • cm^{-2})
TO/TMMP-TMTGE (Original state)	0.0650	2.9621×10^{-6}
TO/TMMP-TMTGE (After scratching)	-0.2762	8.9207×10^{-6}
TO/TMMP-TMTGE (After healing)	-0.0284	4.0785×10^{-6}
TMMP-TMTGE (Original state)	-0.0671	5.9320×10^{-6}
TMMP-TMTGE (After scratching)	-0.3266	1.1064×10^{-5}
TMMP-TMTGE (After healing)	-0.1105	9.0012×10^{-6}
